# Aluminum Enters Mammalian Cells and Destabilizes Chromosome Structure and Number

**DOI:** 10.3390/ijms22179515

**Published:** 2021-09-01

**Authors:** Mirna R. Tenan, Adeline Nicolle, Daniela Moralli, Emeline Verbouwe, Julia D. Jankowska, Mary-Anne Durin, Catherine M. Green, Stefano J. Mandriota, André-Pascal Sappino

**Affiliations:** 1 Laboratoire de Cancérogenèse Environnementale, Fondation des Grangettes, 1224 Chêne-Bougeries, Switzerland; adeline.nicolle@tcbioservices.com (A.N.); emeline@alucancerlab.com (E.V.); stefano@alucancerlab.com (S.J.M.); pascal.sappino@grangettes.ch (A.-P.S.); 2 Wellcome Centre for Human Genetics, University of Oxford, Oxford OX3 7BN, UK; dmoralli@well.ox.ac.uk (D.M.); jjankowska@iimcb.gov.pl (J.D.J.); mary-anne.durin@imm.ox.ac.uk (M.-A.D.); catherine.green@well.ox.ac.uk (C.M.G.)

**Keywords:** aluminium, metal, lumogallion

## Abstract

Chromosome instability (CIN) consists of high rates of structural and numerical chromosome abnormalities and is a well-known hallmark of cancer. Aluminum is added to many industrial products of frequent use. Yet, it has no known physiological role and is a suspected human carcinogen. Here, we show that V79 cells, a well-established model for the evaluation of candidate chemical carcinogens in regulatory toxicology, when cultured in presence of aluminum—in the form of aluminum chloride (AlCl_3_) and at concentrations in the range of those measured in human tissues—incorporate the metal in a dose-dependent manner, predominantly accumulating it in the perinuclear region. Intracellular aluminum accumulation rapidly leads to a dose-dependent increase in DNA double strand breaks (DSB), in chromosome numerical abnormalities (aneuploidy) and to proliferation arrest in the G2/M phase of the cell cycle. During mitosis, V79 cells exposed to aluminum assemble abnormal multipolar mitotic spindles and appear to cluster supernumerary centrosomes, possibly explaining why they accumulate chromosome segregation errors and damage. We postulate that chronic aluminum absorption favors CIN in mammalian cells, thus promoting carcinogenesis.

## 1. Introduction

Several rare elements play essential biological roles in animals and plants. Metal ions are constituents of more than one-third of all cellular proteins, in which they have either catalytic or structural functions [1]. They are vital for the activity of a variety of proteins such as DNA repair and respiratory chain enzymes, and DNA binding transcription factors. However, if, on one hand, specific metal species are essential for cellular functions, on the other hand a disequilibrium in their concentration, intracellular compartmentalization, oxidation state or complex form can lead to cellular toxicity [1,2] and even malignant transformation [3]. Similarly, extraneous metals can deeply perturb the delicate equilibria that allow the proper course of cellular activities and have toxic and/or carcinogenic effects [4,5]. Cytotoxicity or cellular transformation may depend on several factors including the dose and duration of exposure to the foreign metal, with chronic low-dose exposure possibly favoring tumorigenesis [6]. 

Aluminum, the most abundant metal and the third most abundant element of the Earth’s crust, does not take part to any known biochemical process in living organisms. In spite of this, due to its mobilization from minerals, promoted by human activities, aluminum is nowadays a ubiquitous component of human life. Humans are chronically exposed to aluminum through drinking water, vaccines, drugs, antiperspirants, cosmetics, agricultural pesticides and food [7,8]. Major routes of absorption are the skin, the nose, the lungs and the gastrointestinal system [7,8]. As a consequence of continuous exposure, aluminum accumulates in several organs including the lung, bone, liver, kidney, brain and mammary gland [7,9]. High levels of this metal have been associated with diseases such as osteomalacia, microcytic anemia, dialysis encephalopathy, Parkinson’s, and Alzheimer’s [10,11,12,13,14]. Aluminum has also been suggested to be a potential human carcinogen implicated in the etiology of breast cancer [15,16]. The daily application of antiperspirants, that are particularly rich in aluminum salts, has been suggested to contribute to the increased incidence and topological redistribution of breast cancers observed in Western societies [15]. In support of this hypothesis, Linhart et al. (2017) recently reported a statistically significant association between breast cancer risk and frequent use (more than once a day) of underarm cosmetic product (UCP) under the age of 30 [17]. In the latter study, self-reported UCP use also correlated with higher aluminum content in the breast. Experimentally, the carcinogenic potential of aluminum has been evidenced by Mandriota et al. (2016, 2020), by showing that normal mouse mammary epithelial cell models cultured in the presence of AlCl_3_ undergo malignant transformation in vitro and form tumors and metastases in vivo in nude, NOD-SCID and immunocompetent syngenic mice [18,19]. The mechanisms of such transformation are still poorly understood; however, a 2.3 to 3-fold increased number of unique structural chromosome rearrangements was observed in those cells, compared to controls [19]. These findings are in line with previous studies demonstrating aluminum genotoxicity in vitro and in vivo [20,21,22,23].

Chromosome instability (CIN) is the main form of genomic instability leading cells to acquire modifications in chromosomes’ structure, such as deletions, inversions and translocations, and numbers [24]. CIN is observed in the vast majority of solid tumors with nearly 90% of human cancers showing aneuploidy and other chromosomal abnormalities [25]. Such instability is an early phenomenon during tumorigenesis and is associated with poor prognosis and multidrug resistance [26]. One mechanism underlying CIN is defective mitotic divisions. The faithful repartition of the cell genome relies on the bipolarity of the mitotic spindle, which ensures that one copy of each sister chromatid is segregated to each daughter cell. Thus, spindles with more than two poles may lead to massive aneuploidy and cell death. This outcome is generally avoided by cells through strategies such as the clustering of extra centrosomes (the organelles constituting the spindle poles) through which they attempt to preserve bipolar division. However, this coalescence process is accompanied by an increased rate of chromosome segregation errors and aneuploidy [27]. 

In this study we employed Chinese hamster V79 cells, a karyotypically stable cell line frequently used for the assessment of chemical carcinogens in regulatory toxicology, to investigate the impact of aluminum on the structural and numerical stability of chromosomes. We first demonstrate that aluminum, when added to the culture medium at concentrations comparable to those measured in human tissues, enters the cells and accumulates intracellularly in a dose-dependent manner. We then show that, under these experimental conditions, the cells exposed to aluminum exhibit a dose-dependent increase in chromosomes harboring DNA double strand breaks (DSB) and in aneuploidy. These effects seem to be mediated, at least in part, by the perturbation of sister chromatid segregation during mitosis, since, upon aluminum exposure, V79 cells assemble abnormal multipolar spindles and appear to cluster extra centrosomes. Together, our results demonstrate that aluminum concentrations in the range of those detected in human tissues promote CIN in a well-recognized cell model for chemical carcinogesis evaluation, thus providing compelling evidence that aluminum is a human carcinogen.

## 2. Results

### 2.1. Aluminum Entry and Accumulation in V79 Cells

**Figure 1:**
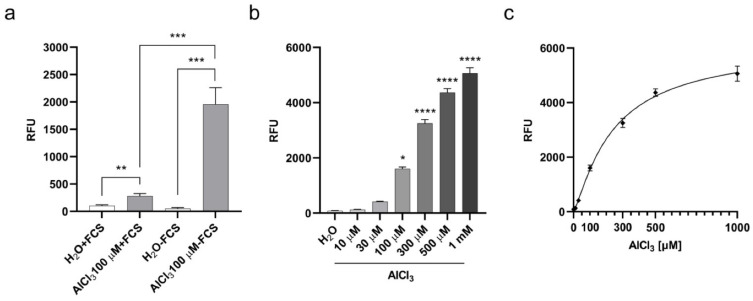
Aluminum absorption in V79 cells upon 3 h of exposure. (**a**) V79 cell aluminum absorption during 3 h in the presence or absence of 10% FCS. Bars represent the mean ± SEM Lumogallion relative fluorescence units (RFU) of five independent experiments each performed in octuplicate. (**) *p*-value < 0.01; (***) *p*-value < 0.001 (Two-tailed Nested *T*-test). (**b**) Dose-dependent aluminum absorption by V79 cells during 3 h, in the absence of FCS. Bars represent the mean ± SEM Lumogallion RFU of three independent experiments each performed in octuplicate. (*) *p*-value < 0.05; (****) *p*-value < 0.0001 (One-way ANOVA with Dunnett’s correction). (**c**) Correlation between AlCl_3_ concentration in serum-free culture medium and cell-associated Lumogallion fluorescence. Non-linear regression curve between medium AlCl_3_ concentration and cell-associated Lumogallion fluorescence intensity. Diamonds represent means ± SEM of the three independent experiments shown in (**b**). R squared = 0.89.

To investigate the potential of cellular penetration and accumulation of aluminum, we adapted a method previously described for aluminum detection in human brain tissues through Lumogallion staining [28] to cultured cells. Lumogallion is a fluorometric chemical with a high affinity for Al^3+^ and forms a 1:1 stoichiometric complex with the soluble ion. Once excited at a wavelength of ca. 500 nm, this complex emits an aluminum-specific fluorescence peaking at ca 600 nm. Lumogallion was not found to bind to Cu^+2^, Fe^+3^, Zn^+2^, Ca^+2^, or Mg^+2^ [28]. We incubated V79 cells with AlCl_3_ 100µM or the same volume of vehicle (H_2_O) alone as a control for 3 h, and then stained them with Lumogallion. Since Transferrin is the main aluminum carrier in the blood [29] and highly abundant in serum, we carried out the incubations either in the presence or absence of 10% Fetal Calf Serum (FCS) to investigate its impact on aluminum incorporation. As shown in Figure 1a, fluorescence emitted by cells incubated with AlCl_3_ was significantly higher compared to fluorescence emitted by control cells in both tested conditions. 

Under serum deprivation, aluminum-specific fluorescence was significantly higher than in the presence of serum (Figure 1a). We next explored the quantitative relationship between AlCl_3_ concentration in the cell culture medium and the resulting cell-associated fluorescence. To this end, the cells were incubated for 3 h with increasing amounts of AlCl_3_ in serum-free medium and specific fluorescence was measured by Lumogallion staining. As shown in Figure 1b, aluminum-specific fluorescence in the cells was dose-dependent, reaching statistically significant higher levels over fluorescence in control cells for AlCl_3_ concentrations equal to or above 100µM. In addition, the increment in cell-associated aluminum-specific fluorescence significantly correlated with the increasing AlCl_3_ concentrations in the culture medium (R squared = 0.89; Figure 1c), confirming the specificity of the Lumogallion staining and the quantitative link between aluminum concentration in the culture medium and its cell-associated fraction. Overall, these data suggest that aluminum, as AlCl_3_ speciation, enters cultured V79 cells, and that a significant amount of the metal accumulates intracellularly during a relatively short exposure time.

**Figure 2:**
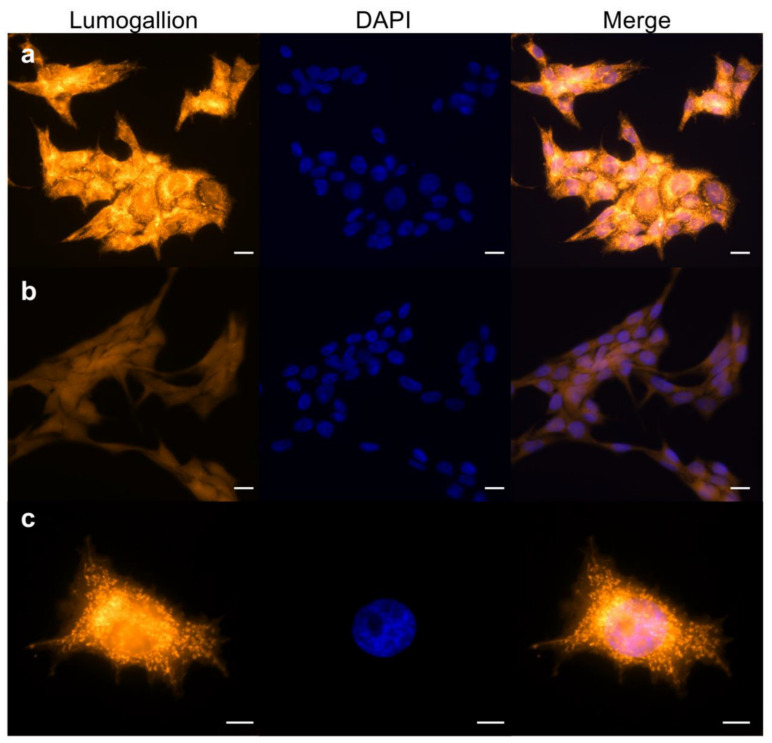
Lumogallion staining of V79 cells exposed to aluminum. The cells were incubated for 3 h with 100 µm AlCl_3_ (**a**,**c**) or the same volume of vehicle (H_2_O) alone (**b**) in serum-free medium. Fixed cells were stained with Lumogallion (orange) and DAPI (blue). Magnification: 40× ((**a**,**b**)); 63× (**c**). Scale bars: 20 µm ((**a**,**b**)); 10 µm (**c**).

To investigate this hypothesis, we analyzed Lumogallion stained V79 cells incubated for 3 h with AlCl_3_ 100 µM—in the absence of FCS—by fluorescence microscopy. Figure 2a shows strong intracellular aluminum-specific fluorescence in the large majority of the cells, with the highest intensity in the perinuclear region. Higher magnification showed a granular-reticular pattern of the staining surrounding the nucleus (Figure 2c). Parallel staining of control cells showed only weak background fluorescence (Figure 2b). Since long-term exposure to AlCl_3_ 10–100µM transforms mammary epithelial cells in vitro [18,19], we wished to extend fluorescence microscopy analysis to mammary epithelial cells incubated for 3 h with AlCl_3_ 100µM—in the absence of serum—and stained with Lumogallion. A staining pattern similar to AlCl_3_-treated V79 cells was observed in human and mouse mammary epithelial cells, including primary human mammary epithelial cells (Supplementary Figure S1A,B). Therefore, when mammalian cells are cultured in the presence of AlCl_3_, aluminum enters the cells where it mainly accumulates in the perinuclear space of the cytoplasm.

### 2.2. Viability of V79 Cells Exposed to Aluminum

**Figure 3:**
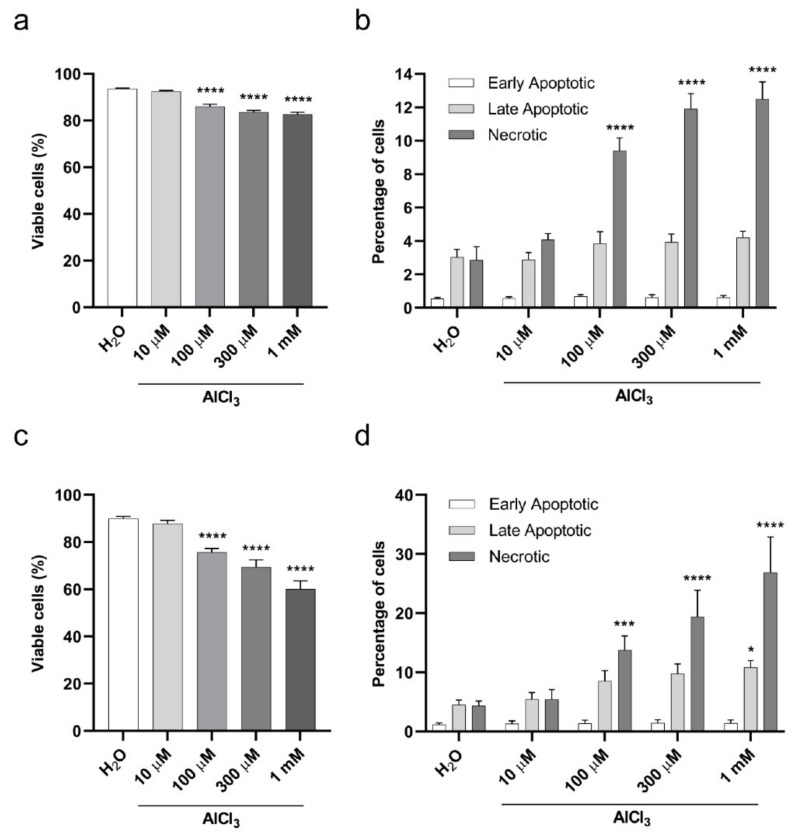
Viability of V79 cells exposed to aluminum by Annexin-V/Propidium Iodide (PI) assay. (**a**) Viability of V79 cells upon 3 h of AlCl_3_ exposure in serum-free medium followed by 17 h of recovery in normal culture medium (without AlCl_3_ addition). Bars represent the mean ± SEM of three independent experiments (H_2_O 93.58 ± 0.28%; AlCl_3_ 10 µM 92.48 ± 0.41%; AlCl_3_ 100 µM 86.07± 0.87%; AlCl_3_ 300 µM 83.53 ± 0.76%; AlCl_3_ 1 mM 82.68 ± 0.84%). (****) *p*-value < 0.0001; (Two-way ANOVA with Dunnett’s correction). (**b**) Percentages of early apoptotic, late apoptotic and necrotic V79 cells in the experiment shown in (**a**). Bars represent the mean ± SEM of three independent experiments. (****) *p*-value < 0.0001; (Two-way ANOVA with Dunnett’s correction). (**c**) Viability of V79 cells upon 24 h of AlCl_3_ exposure (1 h in serum-free medium followed by 23 h with 1% FCS addition without medium change). Bars represent the mean ± SEM of four independent experiments (H_2_O 89.97 ± 0.94%; AlCl_3_ 10 µM 87.82 ± 1.4%; AlCl_3_ 100 µM 75.63± 1.65%; AlCl_3_ 300 µM 69.47 ± 2.97%; AlCl_3_ 1 mM 60 ± 3.56%). (****) *p*-value < 0.0001; (Two-way ANOVA with Dunnett’s correction). (**d**) Percentages of early apoptotic, late apoptotic and necrotic V79 cells in the experiment shown in (**c**). Bars represent the mean ± SEM of four independent experiments. (*) *p*-value < 0.05; (***) *p*-value < 0.001; (****) *p*-value < 0.0001 (Two-way ANOVA with Dunnett’s correction).

Having established that aluminum concentrates in cultured cells in a dose-dependent manner, we wished to assess its impact on cellular viability. To this end, we selected two types of exposure to aluminum based on the Organization for Economic Co-operation and Development (OECD) guidance for the testing of chemicals, namely a short and a continuous exposure [30]. For the short exposure, V79 cells were treated with AlCl_3_ 10 µM, 100 µM, 300 µM or 1 mM or the same volume of vehicle (H_2_O) alone as a control for 3 h in serum-free medium, and Annexin-V/Propidium Iodide (PI) staining was performed after 17 h of cell recovery in normal culture medium (containing FCS), without further addition of AlCl_3_. Starting from a concentration of 100 µM, AlCl_3_ led to a modest but statistically significant concentration-dependent decrease in cellular viability (Figure 3a) and increase in the percentage of necrotic cells (Figure 3b). For the continuous exposure, to maximize aluminum absorption without affecting cell survival by complete serum starvation, cell exposure to aluminum was carried out in serum-free conditions for one hour (the shortest exposure time we tested showing aluminum incorporation by V79 cells; Supplementary Figure S2), followed by 1% FCS addition (without medium change) and an additional 23 h of incubation, for a total exposure time of 24 h. Again, Annexin-V/PI staining showed a dose-dependent, statistically significant (starting from 100 µM AlCl_3_), and more pronounced than observed in the 3 h exposure time, decrease in cell viability (Figure 3c). Under these conditions, exposure to AlCl_3_ 10 µM, 100 µM, 300 µM and 1 mM resulted in 87.8, 75.6, 69.5 and 60% of viable cells, respectively, compared to 90% in H_2_O treated cells (Figure 3c). While the most important fraction of the dying cells were necrotic, apoptosis was also detected in a smaller percentage of cells (Figure 3d). Collectively, these data show a limited, dose-dependent cytotoxicity of aluminum that increased with the duration of exposure.

### 2.3. Aluminum Is a Clastogenic and Aneuploidy Inducing Agent in V79 Cells

**Figure 4:**
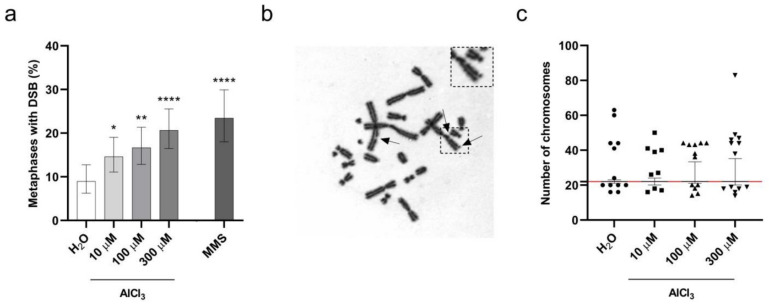
Aluminum induces DSB and aneuploidy in V79 cells. (**a**) Dose-dependent increase in DSB in V79 cells exposed for 24 h (1 h in serum-free medium followed by 23 h with 1% FCS addition without medium change) to the indicated concentrations of AlCl_3_, or the same volume of H_2_O. Parallel treatment with 60 µg/mL MMS for 3 h in serum-free medium followed by 21 h in standard medium was used as positive control. Bars represent the mean percentage ± the Wilson–Brown 95% Confidence Interval. Total number of metaphases analyzed in three independent experiments: H_2_O 301, AlCl_3_ 10 µM 301, AlCl_3_ 100 µM 294, AlCl_3_ 300 µM 305, MMS 192. (*) *p*-value < 0.05; (**) *p*-value < 0.01; (****) *p*-value < 0.0001 (Logistic regression). (**b**) Representative Giemsa stained metaphase of V79 cells exposed for 24 h to 300µM AlCl_3_ as detailed in (**a**). Arrows indicate chromatid breaks. (**c**) Distribution of the number of chromosomes in metaphases of V79 cells exposed for 24 h to the indicated concentrations of AlCl_3_, or the same volume of H_2_O, as detailed in (**a**). The graph shows the median and the percentile range between the 2.5 and the 97.5 percentiles of the number of chromosomes in metaphases. Symbols indicate outlier chromosome number values below the 2.5 or above the 97.5 percentiles. The red line indicates the normal number of chromosomes (22) in a V79 cell. Total number of metaphases analyzed in three independent experiments: H_2_O 299, AlCl_3_ 10 µM 293, AlCl_3_ 100 µM 292 and AlCl_3_ 300 µM 301.

To investigate the impact of incorporated aluminum on chromosomes’ integrity, V79 cells were exposed to AlCl_3_ 100 µM or the same volume of H_2_O vehicle control for 3 h in serum-free medium, and chromosome aberrations were analyzed on metaphase preparations after 17 h of cell recovery in normal culture medium, without addition of AlCl_3_ (that is, the “short exposure” incubation, see previous paragraph). This timing (20 h, starting from the beginning of the treatment) corresponds to approximately 1.5 normal V79 cell cycle duration, as suggested by the OECD guidance [30,31]. Under these conditions, a weak cytotoxicity was observed by Annexin-V/PI staining in AlCl_3_-treated cells, with an approximate two-fold increase in cell death compared to control cells (Figure 3a). Among chromosome abnormalities, aluminum increased the frequency of Premature Chromosome Condensation (PCC), DNA fragmentation and DSB. These frequencies, however, did not reach statistical significance when compared to controls, despite the fact that the *p*-value for metaphases harboring DSB was close to significance (*p*-value = 0.08; Logistic regression). We then thought to extend V79 cell exposure to AlCl_3_ to 24 h (that is, the “continuous exposure”, see previous paragraph), a time equivalent to approximately 1.5–2 normal V79 cell cycle duration, and to expand the range of AlCl_3_ concentrations studied to 10 µM, 100 µM and 300 µM. As mentioned above (Figure 3c), these conditions covered a range of cytotoxicity from almost no cytotoxicity to a maximum of approximately 30% increased cell death for the highest AlCl_3_ concentration tested, corresponding to a four-fold increase with respect to H_2_O treated cells. Thus, according to the requirements set by OECD guidance [30], the cytotoxicity observed in aluminum treated cells was of limited extent. To make our findings statistically reliable, according to OECD guidance, we analyzed a high number of metaphases (approximately 300/condition from three independent experiments). Under these experimental conditions, as for the short exposure time, we detected an increased frequency of PCC in all the AlCl_3_-treated cells, but, again, without reaching statistical significance (Supplementary Table S1). In contrast, we observed a dose-dependent increase in both the number of DSB/metaphase and the frequency of metaphases exhibiting DSB in AlCl_3_-treated cells as compared to control cells (Figure 4a,b and Supplementary Table S1) with 9.0, 14.6, 16.7 and 20.7 percent of the metaphases exhibiting DSB in H_2_O, 10 µM, 100 µM and 300 µM AlCl_3_-treated cells, respectively. 

Logistic regression analysis confirmed that both the number of DSB/metaphase and the frequency of metaphases exhibiting DSB were significantly higher in AlCl_3_-treated cells compared to control cells, and revealed a significant dose-dependent effect of AlCl_3_ (Figure 4a and Supplementary Table S1). We next examined the impact of aluminum on the stability of chromosomal content. V79 cells have a 2n karyotype of 22 chromosomes; therefore, numerical changes in chromosomes were analyzed by examining the distribution and spread of the number of chromosomes in metaphase cells upon aluminum exposure. Again, to make our observations statistically reliable, we analyzed a high number of metaphases (approximately 300 metaphases/condition from three independent experiments). As shown in Figure 4c, 24 h treatment with AlCl_3_ 10 µM, 100 µM and 300 µM induced a dose-dependent increase in the frequency of metaphases with losses and gains of chromosomes as demonstrated by the increasing spread of the percentile range, covering the chromosome number of 95 percent of all metaphases between the 2.5 and the 97.5 percentiles. While in H_2_O-treated cells 95% of the metaphases analyzed had a chromosome number comprised between 21 and 23, the same percentage of metaphases in 10 µM, 100 µM and 300 µM AlCl_3_-treated cells had chromosome numbers comprised between 20 and 24, 21 and 33.4, or 20 and 35.25, respectively. Overall, these data reliably demonstrate that 24 h AlCl_3_ exposure provokes both structural and numerical chromosome alterations in V79 cells.

### 2.4. V79 Cells Exposed to Aluminum Arrest in G2/M Phase of the Cell Cycle

In response to DSB, activation of cell cycle checkpoints ensures the genomic integrity of proliferating cells [32]. As we observed a dose-dependent accumulation of DSB in metaphase chromosomes upon aluminum exposure, we wished to investigate whether this would impact cell cycle progression. In 24 h continuous exposure experiments, PI staining in flow cytometry revealed a significant and concentration-dependent decrease in the percentage of aluminum treated cells in the G1 phase of the cell cycle. This was accompanied by a progressive augmentation of cells in S and G2/M phases (Table 1).

In a parallel analysis of V79 cells treated with 40 µg/mL Methyl Methanesulphonate (MMS) as a positive control or vehicle (H_2_O) alone for 24 h in normal culture medium, MMS caused a dramatic decrease in the percentage of cells in the G1 phase of the cell cycle and, similar to aluminum, a simultaneous increase in the percentage of cells in S and G2/M phases (Table 1). Therefore, both aluminum and MMS caused G2/M accumulation in V79 cells. This is consistent with the lack, in this cell line, of functional p53, required for the activation of the G1/S cell cycle checkpoint in response to DNA damage [33]. Overall, consistent with the observation that aluminum provokes both structural and numerical chromosome alterations in V79 cells (Figure 4 and Supplementary Table S1), these data indicate that aluminum arrests V79 cell cycle progression in G2/M.

### 2.5. Aluminum Induces Multipolar Divisions in V79 Cells

**Figure 5:**
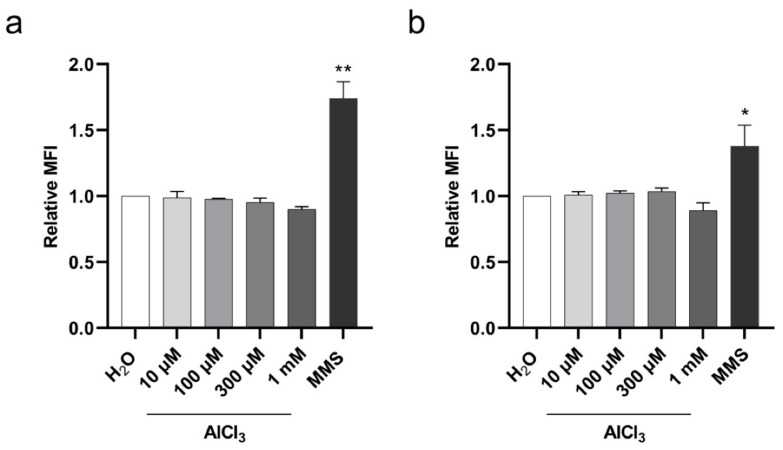
Histone H2AX Ser-139 phosphorylation in V79 cells upon aluminum exposure. The cells were incubated for 3 h with the indicated concentrations of AlCl_3_ or the same volume of vehicle (H_2_O) in serum-free medium. The DNA alkylating agent MMS was used as positive control. γ-H2AX levels were measured by flow cytometry in G1 (**a**) and S (**b**) phase cell populations based on concurrent analysis of DNA content by DAPI staining. Bars represent the Relative Mean Fluorescence Intensity (MFI) (mean± SEM) of four independent experiments. MFI values were expressed as the geometric mean values of the γ-H2AX fluorescence intensity signal of each population of control or treated cells in the G1 or S phase of the cell cycle. Relative MFI values were calculated as the ratio of MFI values of treated cells to the mean of MFI values of control cells. (*) *p*-value < 0.05; (**) *p*-value < 0.01 (Nested one-way ANOVA with Dunnett’s correction).

Having demonstrated a significant increase in DSB upon aluminum exposure, we wished to investigate whether the observed DSB might be the result of a direct DNA damaging effect of aluminum. To this end, we examined Ser-139 phosphorylation of histone H2AX (γ-H2AX), an early cell response to DSB [34], on V79 cells exposed to aluminum for 3 h. The cells were treated with AlCl_3_ 10 µM, 100 µM, 300 µM or 1 mM or the same volume of vehicle (H_2_O) alone as a control in serum-free medium, and the level of γ-H2AX was measured by flow cytometry. Simultaneous DNA staining with 4’,6-diamidin-2-fenilindolo (DAPI) was used to monitor γ-H2AX levels across the cell cycle and exclude DNA damage-independent γ-H2AX on dividing cells in the G2/M phase [35]. As shown in Figure 5, AlCl_3_ did not increase γ-H2AX levels as compared to controls in either G1 (Figure 5a) or S (Figure 5b) phase cells. In contrast, a statistically significant increase in γ-H2AX levels in G1 (Figure 5a) and S (Figure 5b) phase cells were observed upon exposure to MMS, a DNA alkylating agent. These results indicate that DSB accumulation in aluminum-treated V79 cells does not result from an early effect of aluminum, directly damaging DNA. 

**Figure 6:**
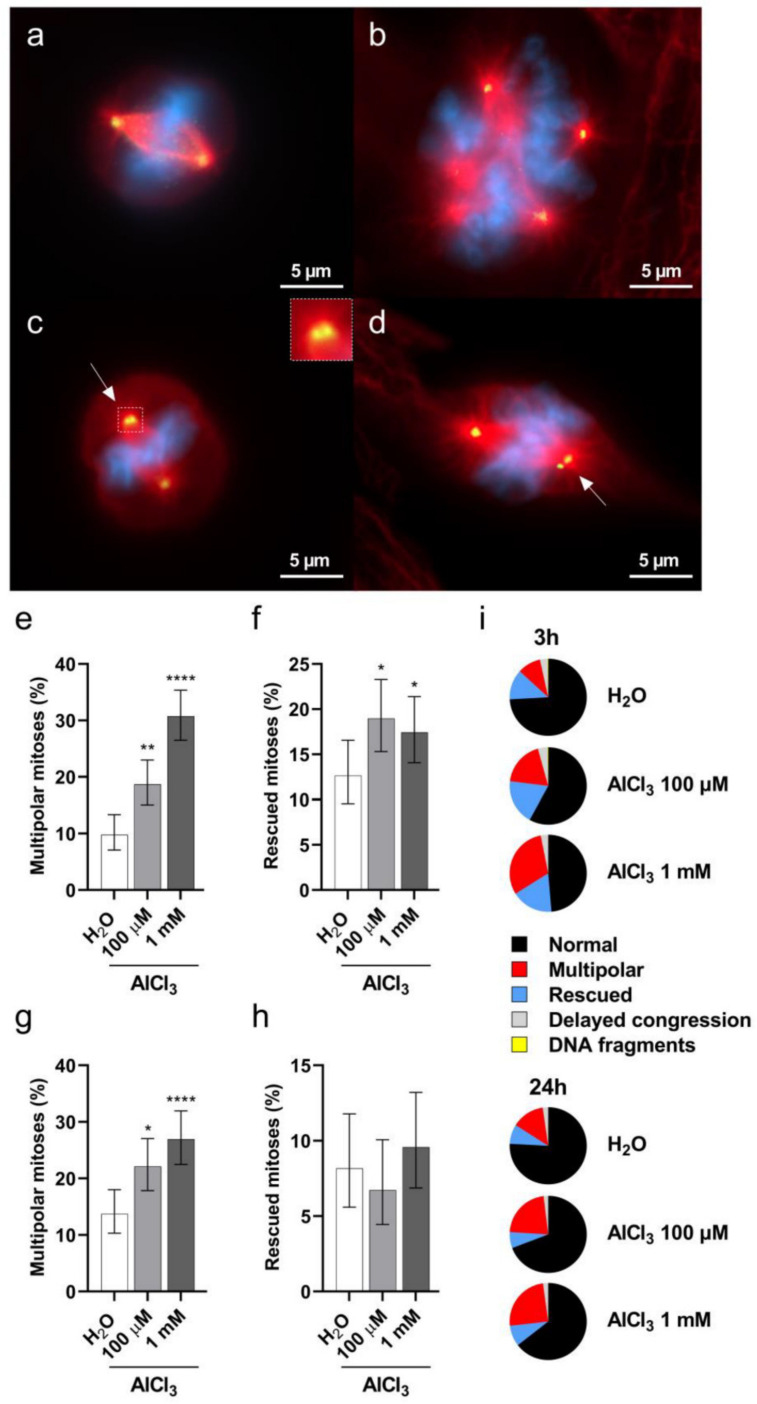
Aluminum induces mitotic spindle abnormalities in V79 cells. The cells were exposed for 3 h to 100 µM or 1 mM AlCl_3_ or an equivalent volume of H_2_O vehicle control. After 17 h of recovery in normal culture medium (in the absence of AlCl_3_) the cells were fixed and stained for DNA with DAPI (blue), for centrosomes with gamma-tubulin (green), and for microtubules with alpha-tubulin (red). (**a**) Representative image of a normal bipolar H_2_O-treated cell at the metaphase stage of cell division. (**b**) Representative image of a multipolar 1 mM AlCl_3_-treated cell at the metaphase stage of cell division. (**c**,**d**) Representative images of rescued 1 mM AlCl_3_-treated cells at the metaphase stage of cell division. The cells are dividing in a pseudo-bipolar manner with two centrosomes at one spindle pole (arrows and inset). (**e**,**f**) Quantification of multipolar (**e**) and rescued (**f**) metaphase mitoses upon 3 h exposure with the indicated concentrations of AlCl_3_ or the same volume of vehicle (H_2_O) in serum-free medium, followed by 17 h of recovery in normal culture medium (in the absence of AlCl_3_). Bars represent the mean percentage ± the Wilson–Brown 95% Confidence Interval. (**g**,**h**). Quantification of multipolar (**g**) and rescued (**h**) metaphase mitoses upon 24 h exposure (1 h in serum-free medium followed by 23 h with 1% FCS addition without medium change) with the indicated concentrations of AlCl_3_ or the same volume of vehicle (H_2_O). Bars represent the mean percentage ± the Wilson–Brown 95% Confidence Interval. (**i**) Proportions of metaphase mitotic abnormalities upon 3 h (**e**,**f**) or 24 h (**g**,**h**) exposure to the indicated concentrations of AlCl_3_ or the same volume of vehicle (H_2_O). Total number of metaphases analyzed in four independent experiments in (**e**,**f**), and (**i**) (3 h): H_2_O 348, AlCl_3_ 100 µM 369, AlCl_3_ 1 mM 413. Total number of metaphases analyzed in three independent experiments in (**g**,**h**), and (**i**) (24 h): H_2_O 306, AlCl_3_ 100 µM 312, AlCl_3_ 1 mM 334. (*) *p*-value < 0.05; (**) *p*-value < 0.01; (****) *p*-value < 0.0001 (Logistic regression).

Previous studies have shown that a prolonged G2 phase of the cell cycle favors the formation of multipolar divisions [36], which are a potential source of aneuploidy and DSB accumulation on mis-segregating chromosomes [27,37]. Having demonstrated both a G2/M cell cycle arrest and aneuploidy in V79 cells following aluminum exposure, we then wished to investigate whether AlCl_3_ treatment would result in an increased frequency of multipolar spindles in V79 cells. To this purpose, V79 cells were exposed for 3 h to AlCl_3_ 100 µM or 1 mM, or to the same volume of H_2_O as a control, in serum-free medium. After 17 h of recovery in normal medium (without the addition of AlCl_3_; see “short exposure” in the text above), the cells were stained for DNA and for gamma-tubulin to visualize mitotic spindle poles. Mitotic cells in different stages of cell division were imaged and analyzed for the presence of multipolarity, delayed congression, lagging chromosomes, chromatin bridges and centrosome clustering. As shown in Figure 6, AlCl_3_ exposure caused a statistically significant increase in metaphase cells with multipolar spindles (Figure 6b,e,i) and in cells appearing to rescue from this state, possibly through the clustering of extra centrosomes (Figure 6c,d,f,i). We also performed a similar analysis upon the “continuous exposure” (24 h) conditions. Again, 100 µM and 1 mM AlCl_3_ treatment led to a significant increase in multipolar metaphase cells (Figure 6g,i). Compared to the 3 h exposure, the continuous aluminum exposure did not result in an increased frequency of rescued cells (Figure 6h,i), possibly because of the lower proliferation potential conferred by the 1% serum conditions of this exposure. Collectively, these data consistently show that aluminum exposure increases the frequencies of multipolar mitotic cells. This raises the possibility that these cells, in an attempt to divide in a bipolar mode, activate the clustering of supernumerary centrosomes. 

## 3. Discussion

The present study aimed at exploring the impact of aluminum on chromosome stability by using a well-established cell model system and an adapted OECD method in carcinogenesis screening, while parallelly providing evidence of the intracellular presence of the metal under the tested conditions. We show that V79 cells cultured in the presence of aluminum in the form of AlCl_3_ do incorporate the metal, as do mammary epithelial cells of both mouse and human origin, including primary human mammary epithelial cells. Our Lumogallion stainings show that aluminum added to the culture medium in the form of AlCl_3_ can enter the cells when these are cultured under standard culture conditions (10% FCS) and to an extent significantly higher when cultured under serum-free conditions. Importantly, normal mammary epithelial cell models were shown to undergo cellular transformation in vitro and form tumors and metastasis in vivo when cultured under comparable conditions, in the continuous presence of AlCl_3_ [18,19,38]. The metal is incorporated by V79 cells in a dose-dependent manner, significantly correlating with the concentration of AlCl_3_ in the culture medium. The correlation was evidenced by Lumogallion staining of exposed cells combined with measurements of emitted fluorescence through a fluorescence microplate reader. These data highlight the specificity of Lumogallion in detecting the intracellular metal and provide a fast and sensitive means of semi-quantitatively evaluating the magnitude of its accumulation into the cells. These findings are in line with previous studies showing a concentration-dependent uptake of aluminum in rat brain endothelial cells exposed to Al-citrate by inductively coupled plasma mass spectrometer quantification of the metal [39]. However, the latter study did not provide information on intracellular localization. Dose-dependent uptake was also observed for aluminum-based adjuvant particles (Alhydrogel) by monocytic-macrophagic THP-1 cells, despite the possibly different mechanism of internalization, given the particulate semi-crystalline form of aluminum and nature of the cell type involved [40]. At present, it is unclear how aluminum crosses the cell membranes. As discussed by Exley and Mold (2015), the route of aluminum transport may depend on its complexation in the aqueous medium at neutral pH [41]. In this context, the Al^3+^ cation may bind to common media constituents and, depending on the size and charge of the complex, it could be transported into the cells through diffusion, ion channels and pumps, or receptor-mediated endocytosis [41]. When serum is present in the culture medium, aluminum will also, and possibly predominantly, bind to Transferrin [29]. Whether this implies that under standard culture conditions—in the presence of FCS—aluminum is mainly internalized through Transferrin Receptor 1 (TFR1)-mediated endocytosis is not known. Our results showing a significant reduction in internalized aluminum upon cell exposure in the presence versus absence of serum may suggest that aluminum forms complexes with serum components leading to different cell transport mechanisms and/or kinetics. Fluorescence microscopy analysis of Lumogallion stained cells showed cytoplasmic labelling with the highest fluorescence intensity in the perinuclear region. This was associated with a granular-reticular pattern of the staining, raising the possibility that aluminum could mainly concentrate in the Endoplasmic Reticulum, Golgi and/or lysosomes organelles. Lysosomes were previously suggested to be the main site of aluminum-based adjuvants storage [40].

Several metals are classified as carcinogenic (group I: Arsenic, Cadmium, Nickel and Chromium (VI)), probably carcinogenic (group 2A: Lead) and possibly carcinogenic (group 2B: Mercury and Cobalt) to humans by the International Agency for Research on Cancer (IARC). Metal carcinogenicity occurs through several mechanisms including oxidative damage of biomolecules, inhibition of DNA repair, alteration of redox homeostasis and disturbance of signal transduction pathways [4,42]. These mechanisms are included among the 10 key mechanisms that characterize human carcinogens according to IARC [43]. 

Aluminum is the most abundant metal on the earth’s crust, yet it has no biological role in any known form of life. However, nowadays all living organisms, including humans, are constantly exposed to this metal. While it is recognized that aluminum at high doses is toxic, especially for the central nervous system [44], low chronic doses are considered to be “safe”. Yet, aluminum has been shown to fulfil several of the 10 key IARC criteria [43]. In particular, it is electrophilic, pro-oxidant and genotoxic and promotes genomic instability [19,20,21,22,23,45]. In this study, we show that aluminum concentrations in the range of those measured in several human tissues cause CIN, the major form of genomic instability, by inducing structural and numerical chromosome alterations in V79 cells, a well-established model for the assessment of chemical carcinogenesis. We observed a significant and dose-dependent increase in the frequencies of chromosomes with DSB and of aneuploid cells deviating from the normal karyotype through chromosomes’ losses and gains. Analysis of cell viability under the same experimental conditions excluded the possibility that these observations were the consequence of excessive cytotoxicity, since the highest aluminum concentration tested in our assays (300 µM) decreased cell viability by only 20% compared to controls. Nor our results could be attributed to changes in the pH of the culture medium, as this was prevented by sodium bicarbonate addition. Furthermore, Lumogallion staining of the cells confirmed the presence of aluminum intracellularly and the correlation between the quantity of aluminum accumulated in the cells and its concentration in the culture medium. This suggests that the dose-dependent clastogenic and pro-aneuploidy effects observed were due to the dose-dependent accumulation of intracellular aluminum. In accordance with our findings are previous studies showing aluminum genotoxicity both in vitro and in vivo [19,20,21,22,23].

Two additional considerations, in our opinion, are important with regard to the concentrations of aluminum used in our experiments. The range we selected is based on the concentrations of aluminum measured in different human tissues where this metal accumulates [7,9], and they do not represent those measured in the blood stream of healthy patients (much lower) or aluminum-intoxicated patients, as Willhite et al. (2014) stated in their report [46]. This is an important point to appreciate the relevance of our data for human health. In addition, it is of relevance that 10 µM aluminum did not significantly affect cell viability as compared to control cells. However, at this same concentration, we observed a significant increase in chromosome DSB. This is possibly the most dangerous situation, as these damaged cells have the potential to survive and propagate karyotypic anomalies. This suggests that low chronic doses of aluminum have the potential to be more harmful than higher doses, making them more likely to cause cell elimination by cytotoxicity. Notably, this reflects the reality of human exposure with low doses absorbed daily through a variety of sources. DSB, if not repaired, may lead to structural chromosome rearrangements [47]. This was recently shown in mammary epithelial cells similarly cultured in the presence of AlCl_3_ and harboring 2.3 to 3-fold more unique chromosome rearrangements than their corresponding controls [19]. Following DNA damage, to repair the lesions, cells activate cell cycle checkpoints causing cell cycle arrest [32]. When analyzed for cell cycle progression upon exposure to aluminum under the same experimental conditions that caused DSB, V79 cells showed a significant decrease in G1 and simultaneous accumulation in G2/M phase of the cell cycle. The lack of proliferation arrest in G1 is consistent with the lack of a functional p53 protein in this cell line [33]. Alteration of cell cycle progression upon aluminum exposure supports the induction of DNA damage by the metal. 

A prolonged G2 phase was shown to favor multipolar spindle assembly during mitosis by centrosome amplification or loss of spindle pole integrity [36,48]. If not corrected, multipolar spindles generate highly aneuploid and non-viable cells. To overcome this problem, extra centrosomes are usually clustered by cells into two poles, a process that, while enabling a pseudo-bipolar division and a viable progeny, favors chromosome mis-segregation and aneuploidy [27,49]. We have shown a significant increase in the frequency of multipolar mitoses in V79 cells exposed to aluminum compared to their respective controls. We also found an important fraction of cells that, while recovering after a short exposure to aluminum, possibly attempt to cluster extra centrosomes. The latter process was not observed under continuous aluminum exposure in low serum, suggesting that either a decreased rate of proliferation and/or the persistent presence of the metal may negatively impact the cells’ ability to correct spindle defects. The significant increase in the rate of multipolarity corroborates our finding of a dose-dependent increase in chromosome gains and losses in cells exposed to aluminum and suggests that this metal may promote chromosome segregation errors and aneuploidy via transient multipolar divisions. In this scenario, aluminum would promote CIN by interfering with the process of spindle assembly, perhaps by increasing microtubules stability [50,51], a phenomenon that affects spindle pole integrity [52] and the ability of the cells to correct kinetochore-microtubule attachment errors [53]. Transient multipolar spindles during mitosis would then lead to chromosome mis-segregation and aneuploidy. In this context, DNA damage may occur primarily on mis-segregating chromosomes, possibly when trapped within the cytokinetic cleavage furrow [37], and this, in turn, would affect cell cycle progression. In support of this hypothesis is the lack of a direct DNA damaging effect of aluminum as evidenced by the absence of a significant histone H2AX phosphorylation upon a short aluminum exposure of non-dividing cells. Mis-segregating chromosomes are an important source of DSB and genomic rearrangements [37,54]. In addition, cells experiencing chromosome mis-segregation and aneuploidy, when entering the subsequent S phase, were shown to undergo replication stress and DNA damage [55]. This triggered their evolution towards complex abnormal karyotypes and CIN [55]. Clearly, numerical and structural chromosome aberrations are mechanistically linked. While the mechanisms through which aluminum affects chromosomes integrity and segregation await further investigation, centrosome amplification, aneuploidy and CIN are a common characteristic of human cancers. Centrosome amplification is an early event in tumorigenesis, has a strong correlation with CIN and is associated with disease progression and poor prognosis [56]. Aneuploidy, observed in 90% of solid human tumors, correlates with metastatic behavior, drug resistance and poor patient outcome [25,57,58]. CIN, the major cause of aneuploidy, drives tumor heterogeneity, thus promoting recurrence, metastasis and resistance to therapy [26,59].

In conclusion, using a validated cell model system and a method for chemical carcinogenesis testing, we have shown that V79 mammalian cells cultured in the presence of AlCl_3_ incorporate aluminum in a dose-dependent manner. Aluminum accumulation causes a concentration-dependent increase in the frequency of chromosomes harboring DSB and in aneuploid cells. We also demonstrate that aluminum exposed cells assemble abnormal multipolar spindles during mitosis, thus suggesting that aneuploidy and structural chromosome aberrations could arise during the passage through these atypical mitotic figures. 

Our data show that concentrations of aluminum in the range of those measured in several human tissues promote CIN in mammalian cells. Given the complete absence of a biological role for aluminum in living organisms, its widespread presence in our daily life, and the accumulating evidence that this metal is not inoffensive to humans, we suggest that special consideration should be given to the long-term consequences of regular low-dose absorption for human carcinogenesis.

## 4. Materials and Methods

### 4.1. Reagents

Dulbecco’s modified Eagle’s medium (DMEM), Phosphate Buffered Saline (PBS), Penicillin/Streptomycin (PS), Glutamine, Insulin, Dexamethasone, sterile water, Gelatin B, AlCl_3_ hexahydrate, sodium bicarbonate, HCl, KCl, PIPES, Methanol, Acetic acid, DPX mountant, Formaldehyde, DAPI, Tween 20, Triton X-100, Staurosporine, Ribonuclease A, Bovine Serum Albumin (BSA), MMS and Colcemid were purchased from Sigma-Aldrich (St. Louis, MO, USA). Tris Base, DMEM F12 Glutamax and Human recombinant Epidermal Growth Factor (EGF) were from Thermo Fisher Scientific (Waltham, MA, USA). FCS and Horse serum were from BioConcept Ltd (Paradiesrain, Allschwil, Switzerland). Lumogallion (4-Chloro-6-(2,4-dihydroxyphenyl-azo)-1-hydroxybenzene-2-sulfonic acid) was from Santa Cruz Biotechnology Inc (Dallas, TX, USA). DAKO Fluorescent mounting medium was from Agilent Technologies Inc (Santa Clara, CA, USA). Fluoromount G was from SouthernBiotech (Birmingham, AL, USA). Giemsa stock solution was purchased from Carl Roth (Karlsruhe, Germany).

### 4.2. Antibodies

Anti-gamma tubulin antibody (ab11316) was purchased from Abcam (Cambridge, UK) and anti-alpha tubulin (PA-5 19489) from Thermo Fisher Scientific (Waltham, MA, USA). PE anti-γ-H2AX antibody and PE mouse IgG1 isotype control antibody were purchased from BD Biosciences (Franklin Lakes, NJ, USA). Alexa Fluor 488 Goat anti-mouse (A11029) and Goat anti-rabbit Alexa 647 (A32733) were from Thermo Fisher Scientific (Waltham, MA, USA).

### 4.3. Cell Culture

V79 cells were purchased from Sigma-Aldrich (St. Louis, MO, USA). The cells were cultured in high glucose DMEM supplemented with 1% PS, 2 mM Glutamine and 10% FCS in a humidified incubator under 5% CO_2_ and 37°C. Namru Mouse Mammary Gland (NMuMG) cells were kindly provided by Prof. R. Montesano (University of Geneva, Switzerland). The cells were grown in high glucose DMEM supplemented with 10% FCS and 1% PS. Michigan Cancer Foundation-10A (MCF-10A) cells were purchased from ATCC (Manassas, VA, USA). The cells were grown in DMEM/F12 Glutamax supplemented with 5% Horse serum, 1% PS, 10 ng/mL EGF, 5 µg/mL Insulin, and 1µM Dexamethasone. Primary Human Mammary Epithelial Cells (HMEC) cells were from Thermo Fisher Scientific (Waltham, MA, USA). The cells were received at passage 1, they were cultured in Humec Ready medium (Thermo Fisher Scientific; Waltham, MA, USA) and used at passage 2.

### 4.4. Lumogallion Staining

For fluorescence microscopy analysis, V79 cells were plated on 0.1% Gelatin B coated cover slips. The day after plating the cells were washed twice with PBS and incubated in serum-free medium with 100 µM AlCl_3_ for 3 h. The cells were then fixed for 10 min in 4% formaldehyde diluted in 0.9% NaCl. After one wash with 0.9% NaCl, the cells were incubated with DAPI solution and subsequently washed three times with 50 mM PIPES buffer pH 7.4. Then, the cells were incubated for one hour at room temperature in the dark with 1 mM Lumogallion diluted in PIPES buffer. Finally, the cells were washed three times with PIPES buffer and mounted with DAKO Fluorescent mounting medium. For quantification of Lumogallion fluorescence, 5 × 10^4^ V79 cells were seeded in black Nunc MicroWell 96-Well Optical-Bottom Plates (Thermo Fisher Scientific; Waltham, MA, USA). On the following day, the cells were washed twice with PBS and incubated with the indicated concentrations of AlCl_3_ or H_2_O vehicle control in the presence or absence of 10% FCS for 3 h. Each treatment was carried out in octuplicate with a blank control consisting of cell-free wells carried out in parallel. Cells and blank controls were then stained with Lumogallion as described above except that DAPI incubation was omitted. Fluorescence was measured two hours later with a microplate reader at 510 nm and 595 nm excitation and emission wavelengths, respectively. 

### 4.5. Cytotoxicity Assay by Annexin-V/PI Staining

V79 cells were seeded in 0.1% Gelatin B coated 24-well plates (Milian; Vernier, Switzerland) in triplicate at the density of 2 × 10^4^ cells/well and incubated overnight at 37 °C. On the next day, the medium was removed and after two PBS washes the cells were incubated with the indicated concentrations of AlCl_3_ or the same volume of vehicle (H_2_O) in serum-free medium for 3 h. Treatments were then removed and the cells washed twice with PBS before adding normal complete medium for additional 17 h. For the continuous 24 h exposure assay, the cells were treated for 1 h in serum-free medium, then 1% FCS was added for the following 23 h without medium change. For both 3- and 24-h exposures, to prevent changes in medium pH due to AlCl_3_ addition, 3 times the molar concentration of NaHCO_3_ to the molar concentration of AlCl_3_ was added to each tested dose. Measurements of the pH under these conditions confirmed the absence of medium pH alteration upon AlCl_3_ addition. Staurosporine at 1.5 ng/mL was used as an internal positive control for apoptosis staining. Cell viability was assessed using an Annexin-V/ PI apoptosis detection kit (Thermo Fisher Scientific; Waltham, MA, USA) according to the manufacturer’s instructions. Stained cells were analyzed on an Attune Nxt flow cytometer. Data analysis was performed using FlowJo software (version 10.7.1). Cells were considered viable if Annexin-V^–^ PI^–^, early apoptotic if Annexin-V^+^ PI^–^, late apoptotic if Annexin-V^+^ PI^+^, and necrotic if Annexin-V^–^ PI^+^.

### 4.6. Metaphase Spread

V79 cells were plated in T25 flasks at the density of 3 × 10^5^ cells/flask. On the following day, the cells were incubated with the indicated concentrations of AlCl_3_ or the same volume of vehicle control (H_2_O) in serum-free medium for one hour. Then, 1% FCS was added to the cells for the following 23 h of incubation, without medium change. To prevent changes in medium pH due to AlCl_3_ addition, 3 times the molar concentration of NaHCO_3_ to the molar concentration of AlCl_3_ was added to each tested dose. During the last hour of incubation 10 µg/mL Colcemid were added to the cultures. As a positive control, cells were treated with 60 µg/mL MMS for 3 h in serum-free medium. MMS was then removed and the cells were grown for additional 21 h in standard medium. Metaphase spreads were prepared as described [60]. Briefly, cells were enzymatically removed from the flasks and incubated 10 min at 37 °C in 0.075 M KCl. After 5 min centrifugation at 200 g, the cells were fixed three times in Carnoy’s solution (3:1 Methanol/Acetic acid), each fixation being followed by 5 min centrifugation at 200 g. Cell suspensions were then dropped on the slides tilted at 45°. Slides were then stained with standard Giemsa (1:20 dilution in 0.1x PBS pH 7.4) and mounted with DPX mounting medium.

### 4.7. Cell Cycle Analysis

V79 cells were seeded on 0.1% Gelatin B coated 24-well plates (Milian; Vernier, Switzerland) in triplicate at the density of 2 × 10^4^ cells/well and incubated overnight at 37 °C. On the following day, the cells were treated for 24 h with AlCl_3_ as described for the cytotoxicity assay. MMS at a concentration of 40 µg/mL was used as positive control in complete medium, together with a corresponding H_2_O vehicle control. The cells were harvested at the end of incubation and washed with 1% BSA in PBS before fixation with BD Cytofix™ Fixation Buffer (BD Biosciences; Franklin Lakes, NJ, USA). Then, the cells were permeabilized with 0.1% Triton X-100 in PBS and DNA content was determined using a solution of 30 µg/mL PI (Thermo Fisher Scientific; Waltham, MA, USA) and 100 µg/mL of Ribonuclease A. Stained cells were analyzed on an Attune Nxt flow cytometer. Data analysis was performed using FlowJo software (version 10.7.1). 

### 4.8. γ-H2AX Staining

V79 cells were seeded in 24-well plates at the density of 4 × 10^4^ cells/well. On the following day, the medium was removed and after two PBS washes the cells were treated for 3 h with the indicated concentrations of AlCl_3_ or H_2_O vehicle control in serum-free medium. To prevent changes in medium pH due to AlCl_3_ addition, 3 times the molar concentration of NaHCO_3_ to the molar concentration of AlCl_3_ was added to each tested dose. MMS at 40 µg/mL was used as positive control. Each treatment was performed in triplicate. At the end of the incubation, the cells were harvested, washed with 1% BSA in PBS (FACS buffer), stained with fixable viability die (Live/Dead fixable Yellow, Thermo Fisher Scientific; Waltham, MA, USA) and diluted 1:200 in FACS buffer for 30 min at 4 °C. After washes with FACS buffer, the cells were fixed and permeabilized with 1× Fix/Perm solution (Transcription Factor Buffer Set, BD Biosciences; Franklin Lakes, NJ, USA), and washed twice with 1× Perm/wash buffer (Transcription Factor Buffer Set, BD Biosciences; Franklin Lakes, NJ, USA). Intracellular staining was performed using a PE anti-γ-H2AX antibody diluted 1:200 in 1× Perm/Wash buffer for 30 min at 4 °C. DNA content was determined by staining the cells with DAPI at 5 µg/mL in 1× Perm/Wash buffer for 10 min at 4 °C. Finally, the cells were washed with 1× Perm/Wash buffer, resuspended in FACS buffer and stored at 4 °C until acquisition, where 8 × 10^3^ cells/technical replicate were acquired and analyzed with Attune Nxt Acoustic flow cytometer. Data analysis was performed using FlowJo software (version 10.7.1). Mean Fluorescence Intensity (MFI) values were expressed as the geometric mean values of the γ-H2AX fluorescence intensity signal of each population of control ortreated cells in the G1 or S phase of the cell cycle. Relative MFI values were calculated as the ratio of MFI values of treated cells to the mean of MFI values of control cells. 

### 4.9. Immunofluorescence

For the morphological analysis of mitotic cells, 1.5 × 10^5^ V79 cells were seeded on 0.1% Gelatin B coated coverslips. On the following day, the cells were washed twice with PBS and then incubated for 3 h with the indicated concentrations of AlCl_3_ or H_2_O vehicle control in serum-free medium. The medium was then removed and after two PBS washes the cells were recovered for 17 h in normal complete medium without AlCl_3_. For the continuous 24-h exposure, the cells were treated for 1 h in serum-free medium, then 1% FCS was added for the following 23 h, without medium change. The cells were then fixed for 5 min in Methanol, blocked for 1 h with PBS-BSA 3% and incubated overnight at 4 °C with anti-gamma tubulin antibody diluted 1/500 in PBS-BSA 3%. After 3 washes in PBS-Tween 20 0.1%, the cells were incubated for 1 h with a Goat-anti-mouse Alexa 488 antibody, stained with DAPI solution, washed and mounted with Fluoromount G. For the representative images of Figure 6, at the end of the treatments the cells were fixed for 3 min in ice-cold Methanol and rehydrated in 0.02 M Tris-HCl pH 7.4, 0.15 M NaCl, 0.1% Triton X-100 (TBS-T). Then, the cells were blocked with TBS-T-BSA 2% for 10 min, followed by 30 min incubation with anti-alpha tubulin antibody diluted at 1 µg/mL in TBS-T-BSA 2%. After 4 washes with TBS-T, the cells were incubated for 30 min with a 1/1000 dilution of Goat anti-rabbit Alexa 647 in TBS-T-BSA 2%. The cells were then washed 4 times with TBS-T and the same procedure described above was applied for gamma-tubulin staining with 2.4 µg/mL of primary antibody and Alexa Fluor 488 Goat anti-mouse diluted 1/2000. After washes in TBS-T, the cells were mounted with Prolong NucBlue (Thermo Fisher Scientific; Waltham, MA, USA).

### 4.10. Image Acquisition

Brightfield and immunofluorescence images were acquired with a Zeiss Axio Imager 2 microscope. Metaphases and mitotic cells were photographed with a Plan-Apochromat 100× /1.4 Oil DIC M27 objective. Lumogallion images were acquired with a Zeiss Axio Observer Z1 with Definite Focus 2 microscope and Axiocam 506 mono camera. Images of 40× and 63× were acquired with a Plan Apochromat 40× /1.4 Oil DIC III (UV) VIS-IR, and with a Plan Apochromat 63×/1.4 Oil DIC III objectives, respectively. Lumogallion excitation was carried out with Colibri 7 led 475, dichroic Quad band Semrock DAPI/GFP/RFP/Cy5, while emission with Bandpass 575–640. The spectrum of Lumogallion emission is broad, ranging from 520 to over 650 nm [40,61] with a peak at around 580 nm. Due to the absence of a longpass emission filter, we used Bandpass 575–640. This filter produced images containing only part of the emitted fluorescence signal. The signal of the stainings, below 575 nm and above 640 nm, was not captured by the monochrome camera. To make the acquired monochrome images as close as possible to the real appearance of the Lumogallion emission spectrum as observed in oculars (where the bandpass filter was not applied), we selected the Lookup Table (LUT) gold of Zen 2.3 software. For each image of aluminum treated cells, the lower and upper values of the data range mapped to the display dynamic range were set to correspond to 99.9% of the pixels. The same upper values were then applied to the corresponding H_2_O control images to allow visual comparison. The lower limit was set to correspond to minimal intensity value. This varied by no more than 4.9% of the data range among images. This data range was mapped linearly (gamma = 1) to the display dynamic range in all images. Images were analyzed with ZEN 2.3 or Fiji-ImageJ software. 

### 4.11. Statistical Analysis

Statistical analysis was performed using GraphPad Prism (version 9.0.0) and R software programs with the tests described in the figure legends. All the results were based on at least three independent experiments. Differences were considered statistically significant when *p*-value was <0.05.

## Figures and Tables

**Table 1:** Cell cycle analysis of V79 cells exposed to aluminum.

	G1	S	G2/M
H_2_O	35.4 ± 1.3	44.5 ± 0.8	14.2 ± 0.3
AlCl_3_ 10 µM	33.5 ± 1.3	46.6 ± 0.9	13.7 ± 0.4
AlCl_3_ 100 µM	30.9 ± 1.1 ^c^	46.8 ± 0.7	16.6 ± 0.6
AlCl_3_ 300 µM	28.9 ± 1.4 ^d^	46.5 ± 0.8	16.9 ± 0.7
AlCl_3_ 1 mM	26.3 ± 1.4 ^d^	48.7 ± 0.6 ^b^	17.7 ± 0.5 ^a^
H_2_O (^+^)	32.3 ± 0.8	45.0 ± 0.5	16.5 ± 0.9
MMS (^+^)	18.3 ± 1.3 ^d^	48.3 ± 0.7 ^a^	23.8 ± 0.8 ^d^

Cell cycle distribution of V79 cells upon 24 h of AlCl_3_—or H_2_O vehicle—exposure (1 h in serum-free medium followed by 23 h with 1% FCS addition without medium change). As a positive control, parallel cultures were treated with 40 µg/mL MMS or H_2_O vehicle in normal culture medium (+) for 24 h. Data represent the mean percentages of cells ± SEM of three independent experiments. (a) *p*-value < 0.05; (b) *p*-value < 0.01; (c) *p*-value < 0.001; (d) *p*-value < 0.0001 (Two-way ANOVA with Dunnett’s correction (AlCl_3_ versus H_2_O) or Sidak’s correction (MMS (+) versus H_2_O (+)) for multiple comparisons).

## Data Availability

The data presented in this study are available upon request from the corresponding author.

## Supplementary Materials

The following are available online at https://www.mdpi.com/article/10.3390/ijms22179515/s1.
