# Supplementary material for: Aluminum Enters Mammalian Cells and Destabilizes Chromosome Structure and Number
Source: Int J Mol Sci. 2021 Sep 1;22(17):9515. doi: 10.3390/ijms22179515 (PMC8431747; doi:10.3390/ijms22179515)
Supplement: Supplementary file 1 [file ijms-22-09515-s001.zip › ijms-1224251-supplementary.pdf]

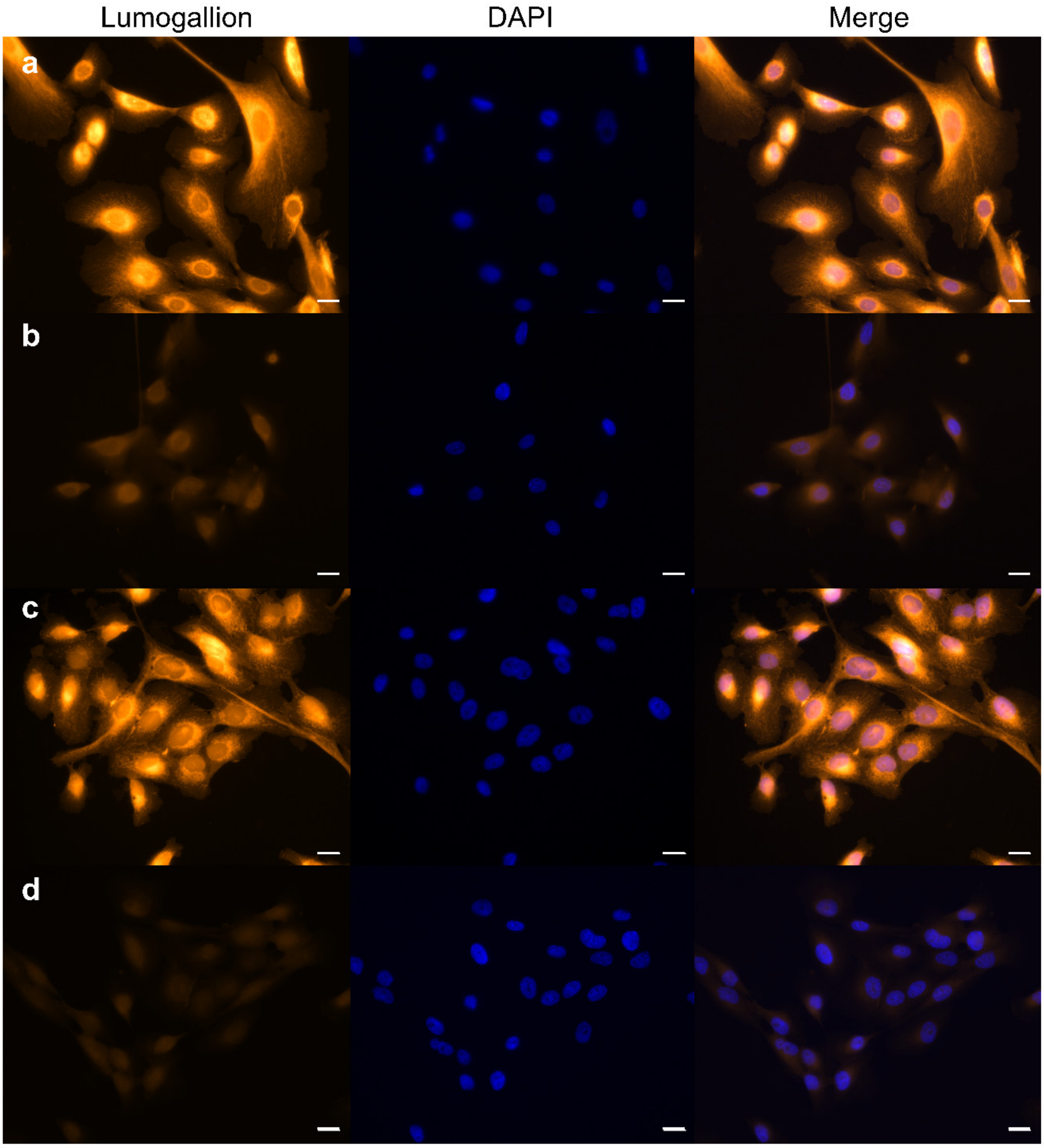
Lumogallion
DAPI
Merge
a
b
c
d

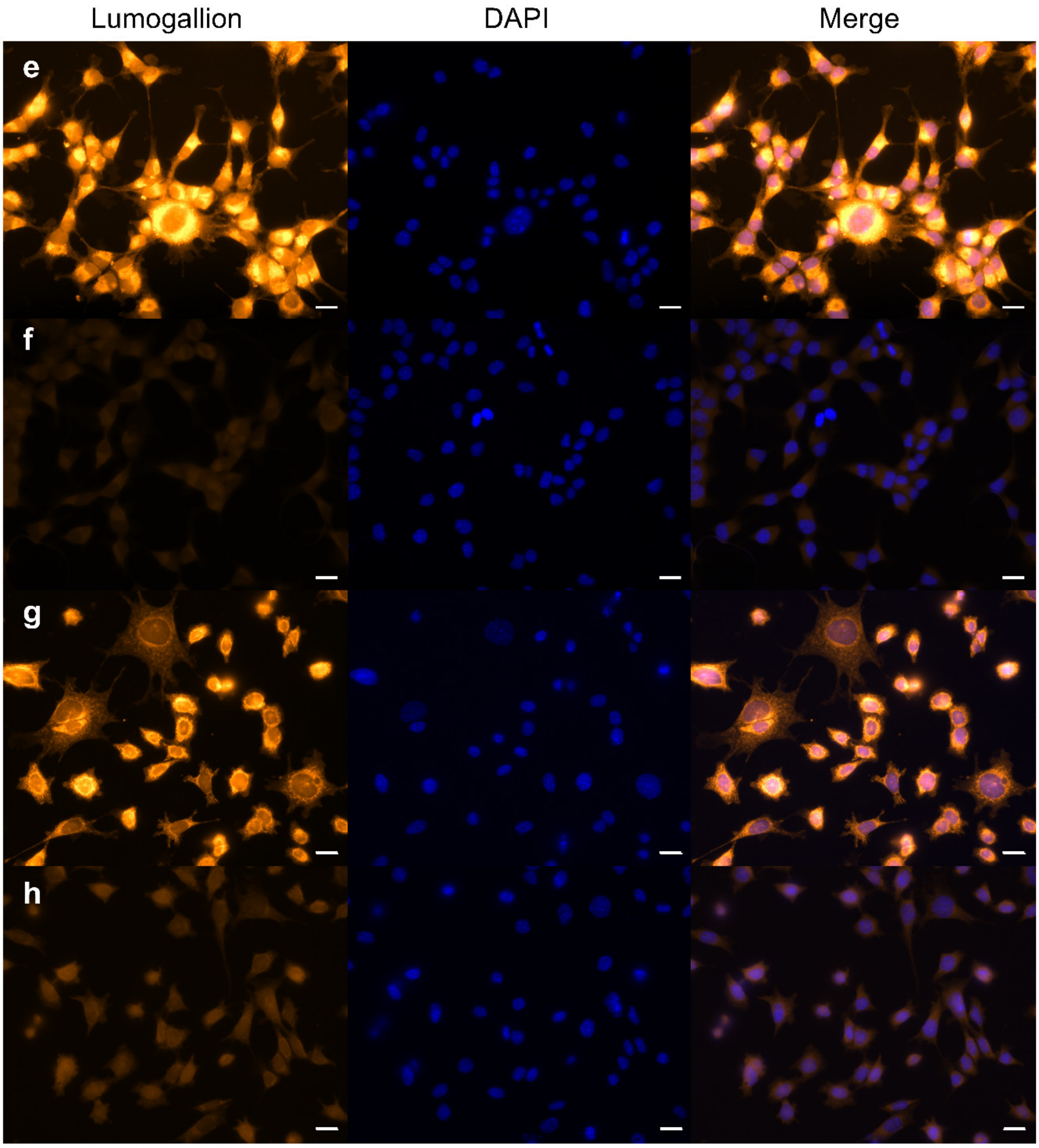


**Supplementary Figure S1. A,B** Lumogallion staining of mammary epithelial cells exposed to $AlCl_3$. Cells were incubated for 3 hours with 100 µM $AlCl_3$ (**a**, **c**, **e**, and **g**) or the same volume of vehicle control ($H_2O$) (**b**,**d**,**f** and **h**) in serum-free medium. Fixed cells were stained with Lumogallion (orange) and DAPI (blue). Primary human mammary epithelial cells (HMEC) (**a** and **b**); MCF-10A (**c** and **d**); NMUMG (**e** and **f**); HC11 (**g** and **h**). Magnification: 40×; scale bar: 20 µm.

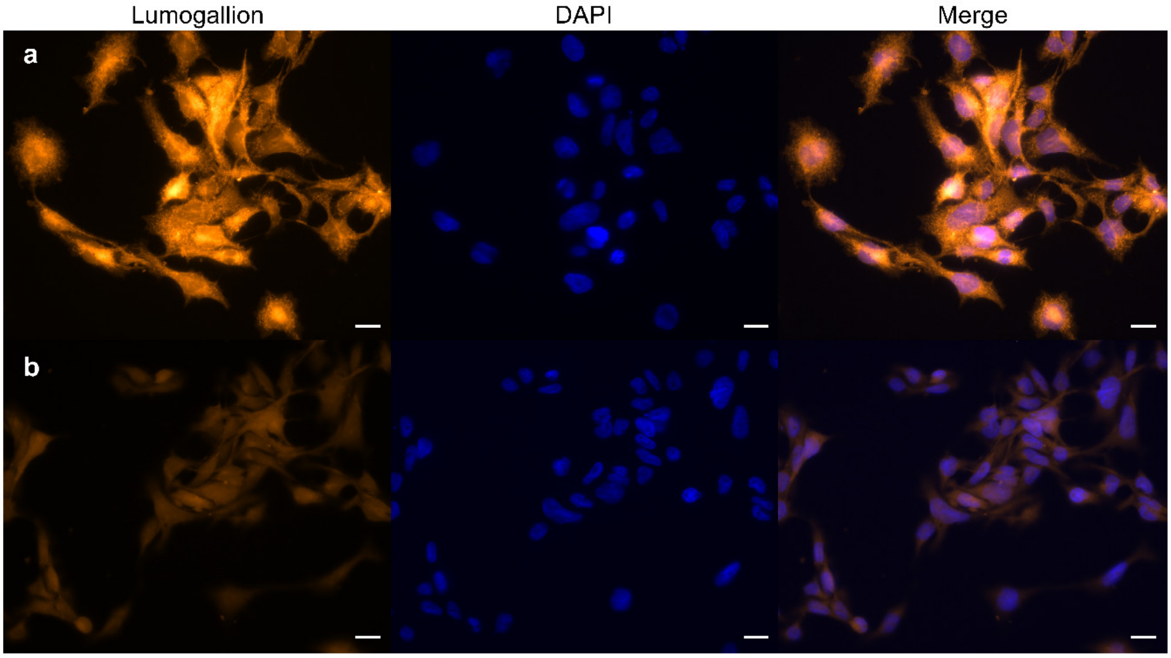


**Supplementary Figure S2.** Lumogallion staining of V79 cells exposed to $AlCl_3$for 1 hour. Cells were incubated for 1 hour with 100 µM $AlCl_3$ (**a**) or the same volume of vehicle control ($H_2O$) (**b**) in serum-free medium. Fixed cells were stained with Lumogallion (orange) and DAPI (blue). Magnification: 40x; scale bar: 20µm.

**Table S1.** Chromosome aberrations in V79 cells exposed 24 hours to $AlCl_3$ for 24 h.

| | **$H_2O$** | **$AlCl_3$ 10 µM** | **$AlCl_3$ 100 µM** | **$AlCl_3$ 300 µM** |
|---|---|---|---|---|
| **PCC** | 2 | 5 | 5 | 6 |
| **DNA DSB** | 27 (**28**) | 44* (**53**) ** | 49** (**57**) *** | 63**** (**69**) **** |
| **Mitotic Slippage** | 5 | 9 | 1 | 2 |
| **Radial** | 0 | 1 | 2 | 0 |
| **Dicentric chromosome** | 2 | 4 | 1 | 3 |
| **Ring** | 0 | 0 | 1 | 0 |
| **Double Minute** | 1 | 5 | 3 | 4 |
| **DNA fragmentation** | 22 | 19 | 21 | 17 |
| **Polyploidy** | 5 | 4 | 7 | 7 |
| **Telomere fusion** | 4 | 3 | 5 | 6 |
| **Pulverization** | 0 | 1 | 1 | 2 |
| **Total metaphases** | 301 | 301 | 294 | 305 |

V79 cells were incubated in the presence of the indicated concentrations of $AlCl_3$ - or vehicle $H_2O$ as a control – for 24 hours (1 hour in serum-free medium followed by 23 hours with 1% FCS addition without medium change). Values represent the total numbers of metaphases exhibiting the indicated abnormalities in three independent experiments. In bold and in brackets are the total numbers of DSB. Metaphases were considered polyploid if harbouring a chromosome number above 30. (*) $p$-value < 0.05; (**) $p$-value < 0.01; (***) $p$-value<0.001; (****) $p$-value<0.0001 (Logistic regression).
